# Enhancing knowledge, attitudes, and practices related to dental caries in mothers and caregivers of children through a neuroeducational strategy

**DOI:** 10.1186/s12903-023-03734-0

**Published:** 2024-01-09

**Authors:** María del Pilar Angarita-Díaz, Elsa Durán-Arismendy, Claudia Cabrera-Arango, Daniel Vásquez-Aldana, Valentina Bautista-Parra, Jessica Laguna-Moreno, Winnifer Mondragón-López

**Affiliations:** 1 GIOMET Group, Faculty of Dentistry, Universidad Cooperativa de Colombia – Campus of Villavicencio, Carrera 35 # 36 99, Villavicencio, Colombia; 2 Scientific Direction. Centro de Competencias Cognitivas y Afectivas, Villavicencio, Colombia

## Abstract

**Background:**

Knowledge, attitudes, and practices related to oral health among parents play a crucial role in shaping oral hygiene and preventing early childhood caries. This study was intended to determine the effect of a neuroeducational strategy in improving knowledge, attitudes, and practices related to early childhood caries among mothers or caregivers of children.

**Methods:**

A quasi-experimental study was conducted, implementing an educational strategy involving 33 mothers or female caregivers of children who met specific selection criteria. The strategy consisted of three key elements derived from neuroeducation: (1) experiment, (2) surprise and play, and (3) learn. Based on the participants’ attendance at the sessions, they were categorized into two groups: those who underwent in-person intervention (G1) and those who received a combined in-person and virtual intervention (G2). The impact of the strategy was evaluated by comparing the participants’ knowledge and attitudes, as well as their children’s plaque index, before and after the intervention (immediate and 6-month impact).

**Results:**

The participants exhibited a favorable and statistically significant effect on the median number of correct answers related to knowledge (G1 immediate effect (IE): *p* = 0.03, 6-month effect (ME): *p* = 0.002; G2 IE *p* = 0.002, ME: *p* = 0.001), and in the children’s plaque index (G1 IE: *p* = 0.003, ME: *p* = 0.003; G2 IE: *p* = 0.033, ME: *p* = 0.003). Furthermore, there was an increase in the number of participants with a high level of knowledge (G1 IE: 41.5%; ME: 75%; G2 IE: 45.5%, ME: 42.9%), and of children with a good level of oral hygiene (G1 IE: 50%; ME: 73.0%; G2 IE: 27.3%, ME: 84.6%). Finally, qualitative interviews revealed a lasting clarity in concepts and sustained knowledge and attitudes at the six-month mark. However, a slightly diminished understanding of the relationship between bacteria, sugar, and caries was observed in G2 group, and some loss of association in the G1 group, at six months.

**Conclusion:**

The implementation of this strategy resulted in significant and lasting impacts on knowledge, attitudes, and practices, especially in the G1 group. Nevertheless, there is a need for further reinforcement of the association between bacteria, sugar, and caries.

**Supplementary Information:**

The online version contains supplementary material available at 10.1186/s12903-023-03734-0.

## Introduction

Dental caries is one of the most widespread noncommunicable diseases worldwide and a matter of public health concern [1, 2]. When present in children under six years of age, it is termed early childhood caries (ECC), characterized by the presence of one or more decayed (non-cavitated or cavitated lesions), missing (due to caries), or filled tooth surfaces on any primary tooth [3]. Untreated caries poses risks to the development of permanent teeth, leading to pain, discomfort, and if it reaches the dental pulp, potential infection, tooth loss, and even systemic diseases. Furthermore, it can have a significant social and economic impact [4].

The disruption of the balance within the oral microbiota present in biofilm is the primary cause of dental caries, mainly due to the prevalence of cariogenic bacteria. These bacteria produce organic acids, which lead to the demineralization of tooth enamel [5, 6]. Various factors contribute to this imbalance, including a high and frequent intake of fermentable carbohydrates such as common sugar (sucrose), and inadequate oral hygiene practices that promote biofilm formation. Consequently, cariogenic bacteria accumulate and metabolize on the teeth [5, 7]. Other predisposing factors for this condition encompass low saliva production, immunological alterations, and even socioeconomic risk factors such as education, income, oral health knowledge, and attitudes among others [8–10].

In Colombia, the prevalence of caries was observed to be 5.89% in one-year-old children, 43.8% in three-year-old children, and 52.2% in five-year-old children. This study also highlights a delayed attendance to dental consultation, where only 58.9% of children under five years of age having been taken for such consultation. Regarding the children’s oral hygiene routines, 15.4% of caregivers indicated that the child performed their hygiene independently, while 39.6% shared the responsibility with the child. Moreover, 74.2% of caregivers had not been provided with guidance on the correct application of toothpaste, and 78.2% had not received instructions on the appropriate amount to use [11].

To address the aforementioned statistics, Colombia has launched the “Soy Generación más Sonriente” (I Am the Smiling Generation) program. Under this initiative, various stakeholders, including local authorities, healthcare institutions, healthcare professionals, academic and research organizations, among others, are involved in educating the population. During dental check-ups and health campaigns, healthcare institutions administer fluoride varnish to individuals under 18 years of age, along with other interventions as outlined in the Comprehensive Health Care Routes [12]. Besides the program, individual interventions are carried out by entities within the general social security healthcare system, particularly focusing on early childhood, childhood and adolescence. These interventions include comprehensive assessments conducted annually, specific protection through fluoride varnish application, prophylaxis, and dental biofilm removal every six months, sealant application as recommended by dentists, and finally, individual, family, and group education [13].

Studies conducted in various countries have shown a significant association between parents’ or caregivers’ limited knowledge about oral health and ECC, highlighting the need for educational initiatives to promote oral health [14–16]. Education is one of social determinant of health since it imparts accurate and reliable knowledge that encourages healthy behaviors and enables the recognition of symptoms related to diseases [17]. Educating people about the causes of dental caries is particularly important, as it helps individuals better comprehend the recommended preventive measures [18]. For example, these preventive initiatives may include efforts to reduce sugar consumption, regular exposure to fluoride and monitoring its levels, initiating and supervising tooth brushing at an early age, understanding the health-disease process, attending dental check-ups, and adopting health-promoting oral hygiene behaviors [18].

 Effective educational strategies that lead to long-lasting learning are essential for improving knowledge and attitudes related to oral health [19, 20]. However, acquiring knowledge about oral health doesn’t always translate into a strong sense of self-efficacy or the adoption of healthy behaviors [21]. As a result, it is necessary to develop strategies based on neuroscience, as this discipline has improved the understanding of the relationship between learning, human cognition, and behavior, thus contributing to neuroeducation [22]. Neuroeducation is an interdisciplinary research field that leverages insights from neuroscience to understand brain function, incorporates principles from psychology to study cognition and human behavior, and applies pedagogical practices to enhance education [23–25]. This interdisciplinary field promotes teaching strategies that involve active participation and integrate elements that optimize information processing, and improve concentration [26].

Among the elements of neuroeducation, “experience” involves stimuli such as sensory stimulation, and simple or complex motor actions. This, in turn, allows for brain reactions and neuronal changes [22]. Another element is “emotions” where the cognitive experience constitutes the sensation. Emotions and feelings play a significant role in consolidating learned contents more efficiently and are responsible for spontaneous reactions and thought processes. They also contribute to decision-making [25, 27]. As part of the emotions, experimentation and play serve as didactic resources that stimulate curiosity and motivation for learning. Experimentation promotes the interpretation, reasoning, and cognitive skills related to a particular subject, while play facilitates the transmission of information and enhances working memory [28, 29].

Neuroeducation has enabled the development of interventions, particularly aimed at students, to enhance information processing, attention, and concentration [30, 31]. When it comes to community-based education aimed at improving knowledge, attitudes, and/or practices, studies have reported strategies based on neuropsychology, cognitive neuroscience, or behavioral sciences [32–34]. Recognizing the importance of incorporating certain elements of neuroeducation to enhance the learning processes, a strategy was devised and implemented to explain the etiology of caries and caries prevention measures. This study aimed to determine the effect of a neuroeducational approach on the knowledge, attitudes, and practices related to preventing childhood caries among mothers and caregivers of children aged one to five years.

## Materials and methods

### Study design and study population

This study was approved by the ethics subcommittee at Universidad Cooperativa de Colombia (No. 55.2019). A quasi-experimental study with a before-and-after design and without a control group was conducted from February to October 2022. This research was conducted at an official public kindergarten catering to children aged 1 to 5, primarily from low to middle- income backgrounds in the city of Villavicencio, Colombia (“Sueños de aprender” [“Dreams of Learning”] kindergarten).

The study sample consisted of 33 participants, which corresponded to a power of 99.9%, indicating a low probability of a type II error in the statistical analysis of the results. This information was calculated using the Epidat 3.1 program (Xunta de Galicia/OPS-OMS), taking into account an expected related mean difference for the plaque index of 1.37, and a standard deviation of 0.39 (before the intervention) and 0.29 (after) [35], with a confidence level of 95%. Participants were included based on the following criteria being consistently met throughout the study: (1) Providing voluntary written consent. (2) Being mothers or female caregivers as the strategy included an activity intended to stimulate maternal sensitivity 3) Being mothers or female caregivers of systemically healthy children (aged one to five years). (4) Not having any intellectual or cognitive disability. (5) Being committed to participating in the strategy activities.

### Study phases

The study encompassed 6 phases: (1) Invitation to participate and signing of informed consent; (2) Data collection from participants and their children via cell phone call (sociodemographic variables, clinical history and oral health knowledge, attitude and practices). Before data collection, interviewer standardization was conducted; (3) Clinical oral examination of children and explanation to caregivers about the “Lift the Lips” technique, which allows for the identification of early signs of caries lesions [36]; (4) Implementation of the educational strategy; (5) Assessment of immediate impact: participants were interviewed via phone call, and clinical examinations of the children were performed (fifteen days after strategy implementation); (6). Measurement of knowledge retention: participants were interviewed via phone call, and clinical examinations of the children was conducted (six months after strategy implementation).

### Educational strategy implemented

The present strategy was designed by researchers in the areas of oral microbiology (MPAD), dentistry (CCA), education (ELDA), and neuropsychology (DVA). The strategy was previously implemented in a pilot study [37], allowing for improving the data collection and intervention in this study. The strategy encompassed moments based on experiences that stimulate different emotions. For this purpose, two meetings were held. The first meeting was held in person for all participants, and the second meeting conducted in person or virtually, depending on attendance. The virtual intervention was carried out using the instant messaging application for cell phones (WhatsApp®). As a result, two groups were formed: the completely face-to-face intervention group (G1) and the mixed face-to-face-virtual intervention group (G2). The learning moments were three: (1) Experience, where efforts were made to induce feelings of motherhood, love, and protection (using sensitizing phrases and aromas) to enhance attention during the viewing of an animated story (video) and experimentation in a simulated microbiology laboratory. (2) Surprise and play, which sought to encourage interpretation and reasoning through the observation of experiment results and involved a game played in person to stimulate attention and concentration before the final moment (only for the G1 group). (3) Learn, which provided instructions on healthy habits [24, 26] (Table 1). The children were consistently present during the intervention.

**Table 1:** Neuroeducational strategy implemented

Sections	Learning moments	Day of meeting	Modality	Target	Activities performed
Introduction	-	1	On-site G1 and G2	Encourage emotions through an evocative phrase about motherhood	Phrase “An act of love is to be there for our children”
Intervention 1	Experience	1	On-site G1 and G2	Stimulate emotions through the memory of motherhood	Scent: baby cologne
Intervention 1	Experience	1	On-site G1 and G2	Explain the caries etiology highlighting the effect of sugar on the microbiota and its relationship with caries	Animated video “The great *Streptococcus* attack”
Intervention 1	Experience	1	On-site G1 and G2	Conduct an experiment explaining the process of tooth demineralization	The experiments were conducted in a simulated laboratory, where participants entered wearing lab coats and caps. Participants took a tooth-shaped chalk and placed it in a tube with an acid solution (vinegar + water). This tube could be taken home so they could observe what was going on
Intervention 1	Experience	1	On-site G1 and G2	Conduct an experiment explaining the presence of bacteria in the oral cavity and the effect of toothpaste on them	In the second experiment, participants collected swabs from the children’s teeth, and with the help of the microbiologist, they inoculated them in bacterial culture medium (Brain Heart Infusion-BHI). Before incubation, four separate dots of toothpaste were added in the center to the surface of the medium
Intervention 2	Surprise and play	2	G1: On-site G2: Virtual	Observe the results of the experiments	In the first experiment they could see how the tooth loses minerals in an acid solution, and in the second, they observed the bacteria on the children’s teeth, and the ability of the toothpaste to inhibit the growth of these bacteria. For the virtual intervention, photos of the results of the experiment were sent to them
Intervention 2	Surprise and play	2	G1: On-site G2: no intervention	Implement a team game on the habits that promote or do not promote tooth decay	Participants were organized into two teams (1. healthy habits or 2. unhealthy habits) to compete by carrying a spoon in their mouth with a ping pong ball to the finish line. The group that finished first was the winning group, the consequence of which was presented through photos of healthy children's teeth or decayed children's teeth
Intervention 2	Surprise and play	2	G1: On-site G2: no intervention	Stimulate a playful attitude	Scent: During the game, the aroma of chewing gum was used
Intervention 3	Learn	2	G1: On-site G2: Virtual	Educate about habits that promote oral health	Through posters and a macro model, participants were instructed on: *Signs of caries *Healthy diet *Proper brushing technique *Frequency of brushing *Amount of toothpaste to be used according to age *Importance of fluoride and its proper use *Responsibility of parents and caregivers in children’s oral health *Attendance to the dentist’s office Videos and posters on the same topics handled in the face-to-face group were sent to G2
Closing of the intervention	-	-	On-site G1 and G2	Recognize the effort for participation in the educational strategy	Delivery of a diploma and an oral hygiene kit for the children, with the phrase of the study “An act of love is to be there for our children”
Closing of the intervention	-	-	On-site G1 and G2	Remember the importance of taking care of children’s oral health	Presentation of the results of the study, and reminder of key aspects for oral health (during a kindergarten parent meeting)

### Measuring the impact of the educational strategy on participants

Before and after implementing the strategy, data was collected on sociodemographic variables and the children’s clinical history. A validated questionnaire (Cronbach’s alpha: 0.82) [38], was also administered to measure the oral health knowledge of parents/caregivers of young children. This instrument consists of 25 questions arranged in 63 items. The topics are associated with (1) dental caries (causes, appearance of initial lesions, how to prevent it), (2) tooth brushing (main purpose), (3) baby teeth (importance and care, information received, age of eruption and onset of oral hygiene), (4) toothpaste (importance, age of initial use, control of quantity), and (5) fluoride (function, knowledge of the presence of systemic fluoride in Colombia). The questionnaire categorized knowledge levels based on the number of correct answers as follows: Scarce (0–25 points), Acceptable (26–50 points), and Good (51–75 points). Additionally, open-ended questions on oral health (knowledge, attitudes and practices) were included (Additional file 1: Appendix A1), and the responses were recorded in audio format. The open-ended questions and the interviewers were calibrated in accordance with the pilot study [37].

Furthermore, the children underwent oral examinations using the knee-to-knee technique [39] using a World Health Organization dental probe, a dental mirror, and artificial light to determine the state of the soft and hard tissues. Oral hygiene was measured using the “modified Silness and Löe” dental plaque index. The percentage of dental plaque was determined based on the number of dental surfaces with plaque, and the level of oral hygiene was classified by the percentage of plaque as Poor (31–100%), Fair (16–30%), Good (0–15%) [40].

### Data analysis

Quantitative data analysis was performed using SPSS version 27.0 (IBM Corp, Armonk, NY, USA). This involved an examination of sociodemographic data and the calculation of the frequencies of participants’ knowledge levels and the plaque index levels of the children. In addition, descriptive analyses (were performed on continuous data variables including the calculation of mean, median, mode, standard deviation, and variance, among others. The statistical normality of the data was assessed using Shapiro-Wilk test, while the homogeneity of variances was determined using Levene’s test. Comparative analyses of the of the knowledge scores and the percentages of the dental plaque index was conducted using the non-parametric Wilcoxon signed-rank test due to the non-normal distribution of the data. A significance level of 5% was used for all statistical tests.

The qualitative data analysis was conducted by the researchers. A verbatim transcription of the interviews collected both before and after the strategy was created and reviewed by the CCA. The text was read and re-read, analyzed, and discussed following inductive thematic analysis, where the information was coded manually by MPAD, and reviewed by ELDA. The participants’ expressions in sentences and lines were summarized. Next, categories and subcategories were developed by identifying patterns in the data and merging similar codes. Finally, relevant quotes were extracted to illustrate each subcategory. The transcription data collected after of strategy, was analyzed using the same categories and subcategories to detect changes in the participants.

## Results

### Sociodemographic characteristics of participants and dental history of their children

Most of the participants in the study were mothers within families who had planned pregnancies and were in the adult life cycle. In G1, the majority of participants had completed high school and a similar percentage had finished university. In G2, most participants had technical degrees.

In both groups, the majority of the children were female and five years old (Table 2), and most had visited the dentist and received treatment with prophylaxis and fluoride application being the most common treatments. In terms of habits, most children brushed their teeth twice a day, and consumed sweets or carbohydrates one to three times a day (Additional file 2: Appendix 2). It’s worth noting that half of the children had dental caries.

**Table 2:** Characteristics of the study population

Characteristic of the population	G1	G2
**Relationship %(n)**		
Mothers	75 (9)	77.3 (17)
Grandmother	25 (3)	22.7 (5)
**Desired pregnancy %(n)**		
Yes	100 (12)	100 (22)
No	-	-
**Pregnancy accepted%(n)**		
Yes	100 (12)	100 (22)
No	-	-
**Life cycle %(n)**		
Youth (22–26 years old)	33.3 (4)	4.5 (1)
Adulthood (27–59 years)	66.7 (8)	86.4 (19)
Older person	0	9.1 (2)
Median of age (IQR)	29.5 IQR (24.5–43.3)	37.0 IQR (33.0-47.3)
**Level of schooling %(n)**		
Primary	8.4 (1)	13.6 (3)
Secondary	33.3 (4)	36.4 (8)
Technical	25 (3)	40.9 (9)
Professional	33.3 (4)	9.1 (2)
**Age of child %(n)**		
One year	0	0
Two years	0	9.1 (2)
Three years	16.7 (2)	0
Four years	25 (3)	27.3 (6)
Five years	58.3 (7)	63.6 (14)
**Sex of child %(n)**		
Female	58.3 (7)	63.6 (14)
Male	41.7 (5)	36.4 (8)

G1: fully face-to-face intervention. G2: face-to-face-virtual intervention

### Effect of the strategy on oral health knowledge

**Fig. 1:**
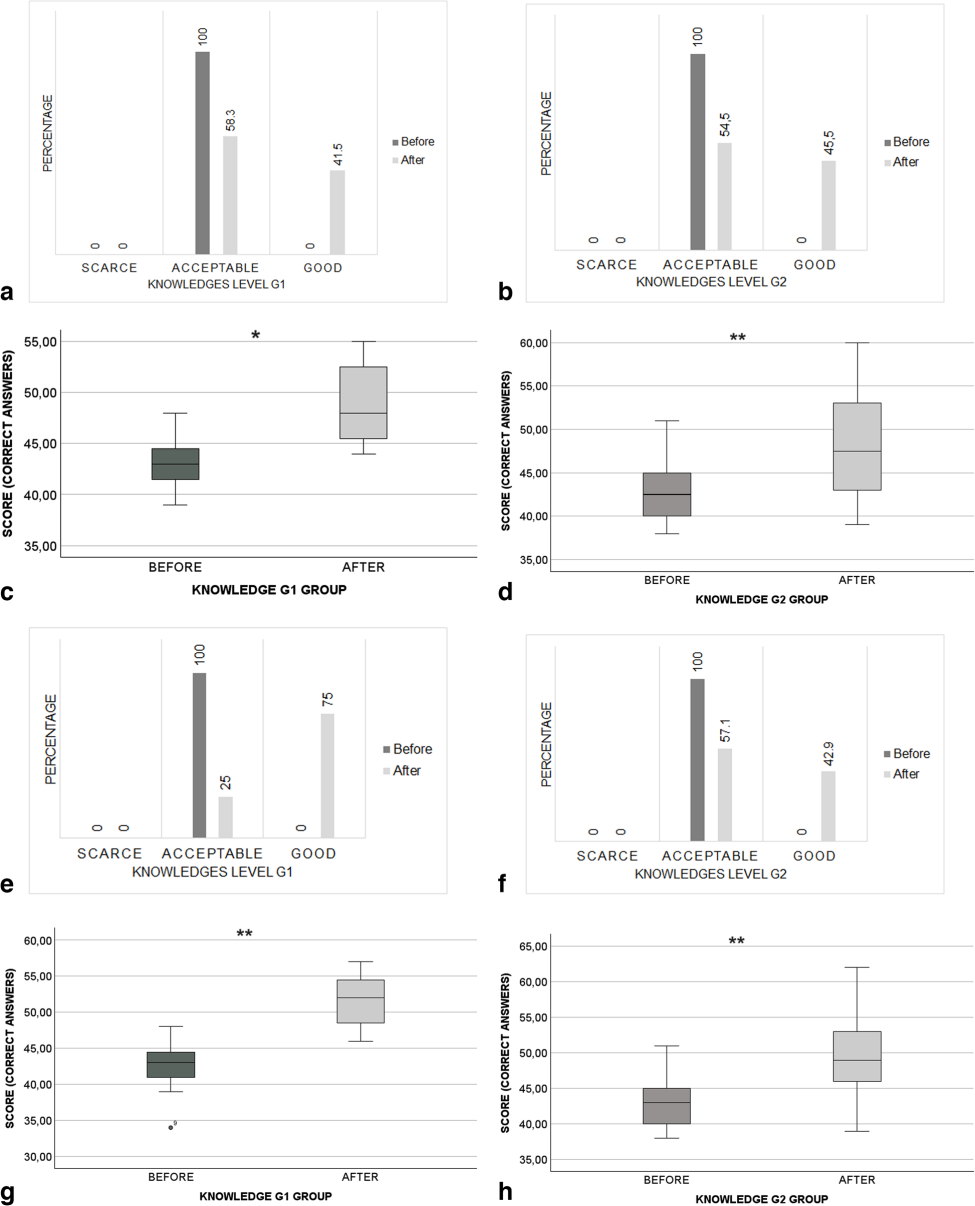
Impact of the strategy on level of knowledge.  Immediate impact (**a**, **b**) Knowledge level of participants in G1 and G2 groups. Level of knowledge about oral health: Scarce level: 0–25 correct answers. Acceptable level: 26–50 correct answers. Good level: 51–75 correct answers. **c**, **d** Score of correct answers in G1 and G2 groups. Wilcoxon test. *p < 0.05 **p < 0.01. Six months post-intervention. **e**, **f** Level knowledge of participants in G1 and G2 groups. **g**, **h** Score of correct answers in G1 and G2 groups

Immediately and 6 months after the implementation of the educational strategy, there was an increase in the percentage of participants who achieved a good level of knowledge, particularly in the G1 group (Before: G1: 0%, G2: 0%; After: G1 immediate effect (IE): 41.5%; G1 6-month effect (ME): 75%; G2 IE: 45.5%, G2 ME: 42.9%) (Fig. 1). This particularly was also detected in the median of correct answers, with a significant improvement in the immediate impact in G1 group (before: 43.0 interquartile range [IQR] [41.3–44.8], after: 48.0 IQR [45.3–52.8], *p* = 0.03) and in G2 group (before: 42.5 IQR [40.0–45.0], after: 47.8 IQR [42.5–53.3], *p* = 0.002) (Fig. 1, Additional file 3: Appendix 3). In terms of the 6-month impact, a significant increase in the median of correct answers was observed again in both the G1 (before: 43.0 IQR [41.3–44.8], after 52.0 IQR [47.3–54.8], *p* = 0.002); and G2 groups (before: 43.0 IQR [40.0–45.0], after: 49.0 IQR [45.0–53.0], *p* = 0.001) (Fig. 1, Additional file 3: Appendix 3).

### Effect of the strategy on oral health knowledge, attitudes, and practices. Qualitative analysis

The educational strategy had an immediate and favorable impact, especially in the G1 group, across various categories, including understanding the etiology of caries (recognizing the presence of bacteria as the cause of the disease and its relationship with inadequate hygiene and dietary habits), early caries detection (identifying white spot as the first sign of the disease), dental visits (emphasizing early visit), responsibility of the person who brushes the teeth (strengthening the commitment to oral health), tooth brushing (promoting the use of a small amount of toothpaste) and emotions (resulting in an increase in positive emotions) (Table 3).

**Table 3:** Categories, subcategories and quotes regarding knowledge, attitudes, and practices before and after the strategy

Category	Subcategory	G1 Phrases			G2 Phrases		
		**Before**	**Immediate impact**	**Impact after six months**	**Before**	**Immediate impact**	**Impact after six months**
1. Concept of caries and its etiology	1.1 Accurate concept of caries associated to etiology	Consequence: *Due to not brushing teeth or due to brushing them incorrectly *Due to eating too much candy *Tooth-damaging bacteria	*Bacteria: -They feed on sweets or food. -High consumption of sweets. -Due to not brushing teeth. *Consequence: -Not brushing teeth or not brushing them properly. -Due to eating too much candy *The candy produces acids and tooth decay appears.	*Bacteria: -They feed on sweets or food. -High consumption of sweets. -Due to not brushing teeth properly. *Consequence: -Not brushing teeth or not brushing them properly -Due to eating too much candy *Candy produces acids and tooth decay appears.	*Consequence: -Not brushing teeth or brushing them incorrectly -Due to eating too much candy *Tooth-damaging bacteria	*Bacteria: -Deteriorate teeth. *Consequence: -Poor hygiene and not going to the dentist -Due to not brushing teeth or brushing them incorrectly -Due to eating sweets	*Tooth-damaging bacteria *Consequence: -Due to not brushing teeth or brushing them incorrectly -Due to eating too much candy
	1.2 Lack of knowledge	1. Does not know	-	-	Does not know	1. I do not remember	-
	1.3 Misconception	*Rotten tooth *Bad breath *Black tooth *Tooth pain	*When the tooth turns black	*Starts when acid is ingested	*Tooth pain *Black spot. *Hole *Bad breath *Hereditary disease *Tartar and crust	*Black spot *Pain in the teeth	*Generates bad odor *Starts with a black spot
2. Sign of caries	2.1 Early sign	*White spot	*White spot *White spot that turns yellow or forms a hole.	*White spot *White or yellow stain *Coloration change	*White-to-yellow stain	*White spot	*White or yellow spot
	2.2 Lack of knowledge	* Does not know *It’s something ugly *Red gum *Bad odor *When told by the dentist	*Smells bad	*Changes in the gum	*Yellow teeth *Bleeding in the gum *Stains and dirt	*Stains on teeth *Yellow teeth *Bringing to follow up	*Inflammation *Bad odor *Spots appearing *When told by the dentist
	2.3 Late sign	*Black or brown stain *Pain in the mouth *A hole starts to appear	*Pain in the teeth	*Pain in the teeth *Black or brown stain *Stain with hole	*Pain in the teeth or in the mouth *Hole *Black spot *When it looks dark	*When the tooth is pitted *Pain in the teeth *Dark teeth *Black spot *Some indentation on tooth	*Black teeth *Pain in the teeth. *Cracks in the teeth *Black or brown stain
3. Effect of candy on tooth decay	3.1 Association between sugar and caries-producing bacteria	-	*Sugar gives energy/food to bacteria *Sweets are associated with bacteria and damage the tooth.	*It is like a bacterium	-	*The sweet forms more bacteria *It causes bacteria to accumulate on the teeth.	*Bacteria stick to the teeth and damage them
	3.2 Association of sugar with caries without linking it to bacteria	-	*Sweets stay in the teeth and cause cavities *Sugar helps to promote tooth decay	*They produce acids that damage the teeth. *Deteriorated teeth *Formation of dental plaque *They help tooth decay to form	*Produces caries by forming cavities. *Sugar eats fluoride out of teeth	*Deteriorates the tooth *Accumulates/adheres and helps tooth decay to form *Begins to give stains and bad breath *Poison for teeth	*It pits the tooth *Consuming too much is bad *It accumulates in the teeth and damages/eats away at the teeth. *Decalcifies
	3.3 Unawareness	*Does not know *Soda pop causes the stains *The candy stays on the gums or teeth.	*If there is no good cleaning, sugar accumulates.	*Does not know	*Does not know *I know they are bad	*It is formed as a small rock that eats the tooth *Does not know	*It is formed as a small rock that eats the tooth *Does not know
	3.4 Lack of knowledge	Not applicable	*Does not remember	*Does not remember	Not applicable	*Does not remember	*Does not remember
4. Actions that prevent caries development	4.1 Correct preventive action	*Brushing teeth: -Good brushing *Decreasing the intake of sweets. *Attending the dental consultation *Good nutrition *Take your child to the dentist from an early age.	*Visit to the dentist *Small amount of sweets *Teaching how to brush teeth. *Dental floss *Apply fluoride *Brushing teeth: -Good brushing -Constant *Good nutrition	*Visit to the dentist *Good tooth brushing *Consuming small amounts of sweets *Flossing	*Brushing teeth: -Regularly -3 times a day -Good brushing *Not eating sweets *Flossing *Eating healthy *Visiting the dentist	*Flossing *Visit to the dentist *Avoiding eating sweets *Daily brushing, 3 times per day *Healthy eating *Care for baby teeth	*Brush well when eating sweets *Brushing teeth: -3 times a day -With toothpaste and brush *Flossing *Eating small amounts of sweets
	4.2 Confusion about the action	*Brushing teeth: -Every day *Not eating a lot of flour	*Mouthwash *Good hygiene	-	*Brushing teeth: -Once a day -In the morning -Every day *Doing treatments	*Brushing teeth: -Every day -Good brushing -Good hygiene *Sealants	
	4.3 Inappropriate action	-	-	-	*Washing with boiled water	*Brushing at all times with baking soda.	
5. Visit to the dentist	5.1 Early visit	*Follow up *Preventing diseases *To learn *Every six months *During the growth and development program *When the first teeth came in *When I was one year old	*Every six months *Every three to six months *Every six months or sooner when there is any problem *To get good advice *Protects teeth	*As follow up *Every six months	*Follow up *Every six months *If there are no cavities, every six months	*Every three to six months *Every six months *For follow up *As prevention	*Every six months *For follow up *When going to growth and development
	5.2 Lack of knowledge	*Does not remember when they took it *Take it every month or every two months	-	-	*Every three months	*Every time the dentist says so *When necessary	*Takes her every month *Every three months
	5.3 Late visit	*The parents don’t have time *When spots are visible *When there is discomfort or pain. *Every year *Has not taken them *When the dentist says	*I took them for the first time during this activity.	*Every year *I have only taken them during the study activity *Pain	*When there is pain *When you see spots or black spots *Every year *I haven’t taken her for a while *They have not taken them	*Every year *To remove a tooth *Because of the pandemic they have not taken him *They took him for the first time during the study.	*Due to an emergency
6. Responsibility of person who brushes the teeth	6.1 Commitment to oral health	*The grandmother teaches them *The parents *The mother *The father *The grandmother *Whoever is in charge of the child	*The father and the mother *The mother *The person in charge *The grandmother	*The father and the mother *The mother *The person in charge	*The mother *The parents *The parents, the grandmother. *Whoever is in charge of the child	*The mother *The parents *The grandmother *Whoever is in charge of the child	*The mother *The parents *The grandmother
	6.2 Shared responsibility	*Children brush and parents reinforce them	-	-	-	-	-
	6.3 Lack of clarity	*Teaching to brush	-	-	-	-	-
	6.4 Delegation of responsibility	*Delegate to the child the responsibility of brushing the teeth	-	-	*The siblings	-	-
7. Tooth brushing	7.1 Small amount of toothpaste	*Uses small amount of toothpaste	Amount of toothpaste: *The tip of the brush *Like a grain of rice *The nail of the little finger *One pea kernel	Amount of toothpaste: *The tip of the brush *Like a grain of rice *The nail of the little finger	Amount of toothpaste: *Two drops of toothpaste *The pinky fingernail. *Uses a small amount *Uses the measure of a grain of rice.	*Uses a small amount of toothpaste *Uses about as much as a grain of rice *Uses the measure of a grain of rice of the fluoride toothpaste. *Uses a dot of toothpaste	*Uses about as much as a grain of rice *Uses about as much as the pinky fingernail
	7.2 Confusion/ Unclear	-	-	-	-	*Uses different amounts of toothpaste	1. Uses a small amount of fluoride-free toothpaste 2. Uses a small amount
	7.3 Lots of toothpaste	Amount of toothpaste *Half of the toothbrush *The whole toothbrush	-	-	Amount of toothpaste: *The whole toothbrush *Half of the toothbrush	Amount of toothpaste: *Half of the toothbrush *The whole toothbrush *Less than half of the brush	*All brushing with fluoride cream *Half of the toothbrush
	7.4 Other	*Mouthwash *Dental floss	-	-	-	-	-
8. Emotions	8.1 Positive	*Anxiety: -Doesn’t have any -Has few *Motivates: -By educating -By buying nice toothbrushes *Likes to brush	*Anxiety: -Doesn’t have any -Has little *Before, he would get nervous, but now he is more confident. *He was not afraid during the activity *He was calm during his first time Motivates: -He tells her that she looks pretty with clean teeth. -With videos	*Anxiety: -Doesn’t have any - Has little *They were scared, but they felt good *They are calmer *Motivates: -Teaches and explains how to care for teeth -Saying they will have a nice smile -With videos of children with cavities -With the example of the parents -To make it smell good	*Anxiety: -No anxiety *Motivates: -Singing the Colgate song -Brushing their teeth beside her -Teaches them to brush *Likes to brush *She takes the initiative	*Anxiety: -No anxiety *Motivates: -To have a nice smile and clean teeth. -Teaching how to brush teeth *They ask to have their teeth brushed	*Anxiety: -Doesn’t have any -She was calmer during the study *Motivates: -Explaining what is happening -Inviting them to brush their teeth with you -Indicates that brushing kills the little bugs -Playing videos of dolls -Singing the Colgate song
	8.2 None	*Doesn’t know if it gives them anxiety *Does not need to motivate	-	-	-	*She cried a little and was soothed with stickers.	-
	8.3 Negative	*Very nervous about everything *They are scared when they are at the dentist’s office *Motivates: -By reprimanding -By threatening	-	-	Anxiety: *She starts to cry *She is scared Motivates: -Does not like to brush -Saying they have dirty teeth -By reprimanding -By threatening	*Anxiety: -They have it -They get nervous -They get scared -She cries and screams *Motivates: -Telling them that if they don’t brush their teeth, they get ugly.	*Anxiety: -They have it -Not all dentists have the same patience. Motivates: -Threatens them saying that they will permanently have a grimace -Threatens to take him to the dentist.

Six months after the implementation of the educational strategy, the G1 group showed a lasting retention of information in the categories related to the etiology of caries, the responsibility of the person who brushes the teeth, tooth brushing and positive emotions. However, in the category of early caries detection, it was observed that participants in the G2 group and some in the G1 group had forgotten this concept (Table 3).

In the category related to the effect of sweetness on caries, it was found that the effect was somewhat lower compared to the other categories. Few participants were able to associate the effect of sugar with the increase of cariogenic bacteria and its role in causing caries. Finally, in the category of actions that prevent the appearance of caries, it was detected that most participants knew about this topic before the intervention, and there were not many significant changes in their understanding (Table 3).

### Effect of the strategy on children’s plaque rate

**Fig. 2:**
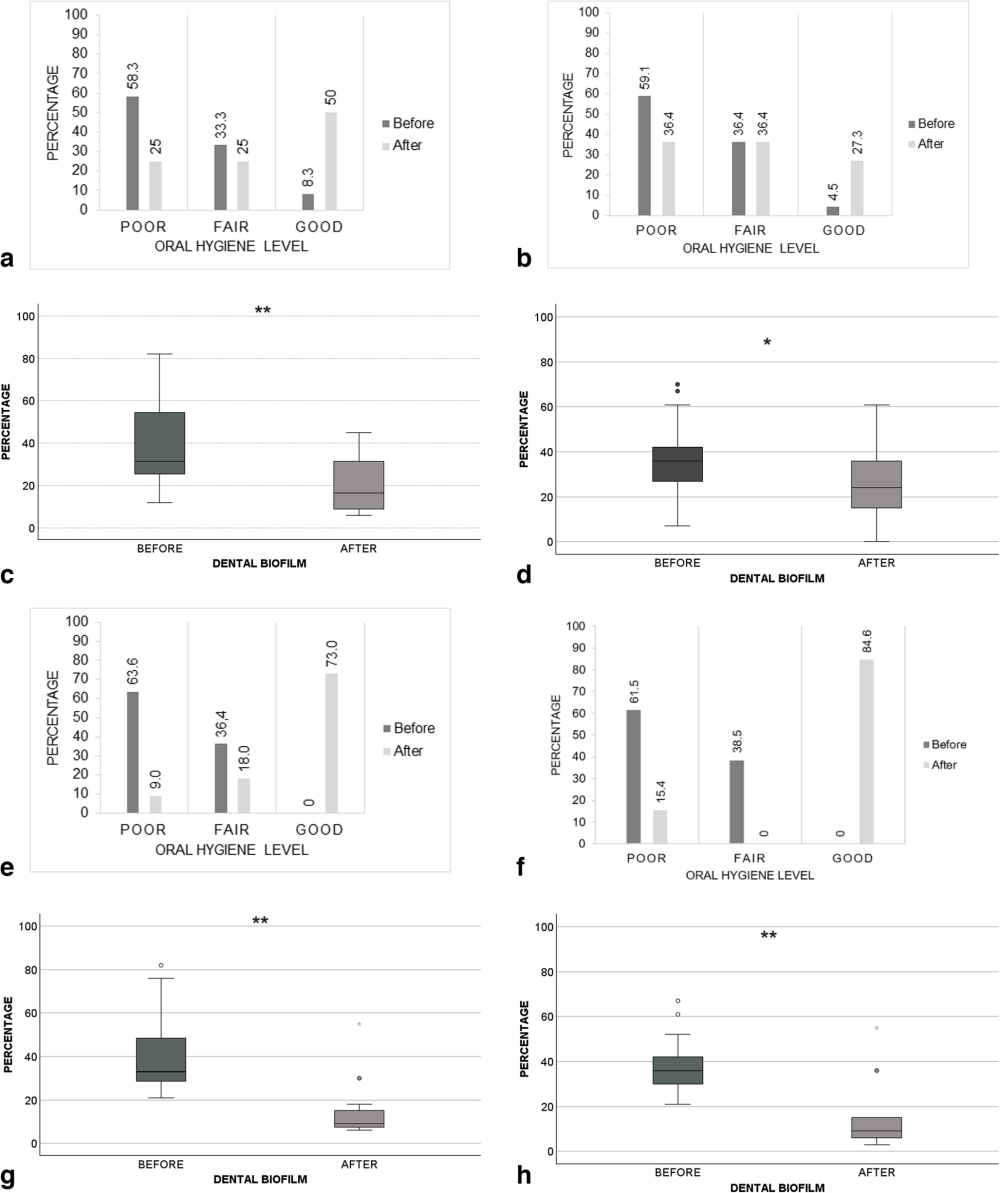
Impact of the strategy on dental plaque index of children’s plaque rate. Immediate impact (**a**, **b**) Level of oral hygiene in G1 and G2 groups. Level of oral hygiene: Poor 31–100%, Fair 16–30%, Good 0–15%. **c**, **d** Percentage of dental plaque in G1 and G2 groups. Wilcoxon test. *p < 0.05, **p < 0.01. Six months post-intervention. **e**, **f** Level of oral hygiene in G1 and G2 groups. **g**, **h** Percentage of dental plaque in G1 and G2 groups

Immediately after the intervention and at the 6-month follow-up, an increase in the percentage of children with a good level of oral hygiene was detected in G1 group (before IE: 8.3%, after IE: 50%; before ME: 0%, after ME: 73%) and G2 group (before IE: 4.5%, after IE: 27.3%; before ME: 0%, after ME 84.6%) (Fig. 2). Furthermore, the immediate impact of the strategy resulted in a statistically significant decrease in the percentage of the plaque index in G1 (before: 31.5% IQR [14.7–31.5], after: 16.5% IQR [6.0–16.5], *p* = 0.003) and G2 (before: 36.0% IQR [26.3–43.5], after: 24.0% IQR [14.3–36.8], *p* = 0.033) (Fig. 2). The 6-month impact also showed a significant decrease in G1 (before: 33.0 IQR [27.0–61.0], after: 9.0 IQR [6.0–18.0], *p* = 0.003) and G2 (before: 36.0 IQR [30.0–47.0], after: 9.0 IQR [4.5–15.0], *p* = 0.003) (Fig. 2).

## Discussion

Before implementing the strategy, it was observed that all participants had an average level of knowledge about oral health, with the majority being unaware of the etiology of caries and exhibiting significant misconceptions in key concepts about oral health (early signs of caries, first visit and frequency of dental visits, amount of toothpaste, and responsible individuals for brushing teeth). Additionally, the majority of the children had poor oral hygiene, and half of them had early childhood caries. These findings highlight a persistent lack of oral health knowledge in the population, as well as insufficient educational and communication actions that really impact and promote healthy practices. This aligns with reports from the latest ENSAB and other studies carried out in Colombia [11, 12, 41].

 The strategy implemented in both the G1 and G2 groups had a positive impact on the knowledge and attitudes of participants, and on the oral hygiene index of their children. The learning achieved in this study could have been influenced by the stimulation of positive emotions and feelings, which play a role in cognitive processes such as attention, memory, motivation, perception, and decision-making [22].

The stimulation of maternal sensitivity, was a crucial element of the activity. In addition to improving knowledge, it also led to a reduction of threatening phrases related to oral care and increased the children’s interest in brushing and oral examinations. This finding confirms what has been demonstrated in other studies regarding the impact of positive parental attitudes on children’s oral health and self-care [42, 43]. For example, a study by Nepaul and Mahomed in 2020 found an association between parental knowledge and positive attitudes, which translated into children displaying happier attitudes toward tooth and tongue brushing [42].

The experimental component of the strategy, which included demonstrating the presence of bacteria on the teeth and the effect of toothpaste in reducing them, played a significant role in the positive results, especially in reducing dental biofilm in children. Several studies have reported the impact of demonstrating the presence of dental biofilm and bacteria in the oral cavity on the oral hygiene practices of participants or their children [35, 44]. For example, a study by Thomson et al. in 2022 showed that implementing oral hygiene instructions along with microbiological explanations and demonstrations increased adherence to hygiene practices and improved understanding of the relationship between oral bacteria and dental disease among pregnant patients [44].

The play component, which aimed to engage participants’ attention and motivation before the learning moment, may have contributed to the face-to-face group’s (G1) higher level of knowledge. This result is likely because play stimulates the production of dopamine, increasing curiosity, attention, and motivation to learn, and enhances the transmission of information between the hippocampus and the prefrontal cortex [45]. Play has been used in different studies, especially with children, to improve knowledge about oral health and hygiene [46, 47]. For instance, a study by Sharma et al. in 2021, which involved a game combined with didactic cards, showed a more significant impact on the oral hygiene index compared to a conventional strategy, in children aged seven and 12 years [47]. However, it is essential to note that G1 has more participants with a professional background compared to the G2 group, which might have also facilitated greater learning.

In our study, we observed not only the immediate impact but also a good retention and even improvement in participants’ knowledge, attitudes, and practices, as well as in the oral hygiene of the children, six months after the intervention. This sustained and enhanced effect has been demonstrated in interventions with psychoeducational characteristics. These interventions do not only enhance knowledge, but also positively influence behavioral intentions, which “are predictors of behavior and are determined by the individual’s attitude and normative social forces to perform the behavior” [32, 33]. For example, a study based on the self-determination theory achieved significantly greater persistence in dietary behaviors, oral hygiene, and avoidance of sharing eating utensils with their children among mothers of one to four-year-old children, compared to a control group [33, 34]. Another strategy based on the theory of planned behavior, led to a significant increase in oral self-care in mothers of children between one and six years of age, both immediately, and at three and six months post-implementation, compared to a control group. In addition, at the six-month follow up, there was a significant increase in the number of children brushing their teeth twice daily [34].

Although the positive outcomes were observed immediately and at the six-month follow-up, there was notably a lower understanding of the white spot as the first sign of caries, and a weaker connection between the presence of bacteria, high sugar consumption, and the development of caries primarily within the mixed intervention group (G2). These results highlight the importance of the components used in fully face-to-face participation, such as the play. Nonetheless, after 6 months, the results for G1 and G2 in terms of the oral hygiene index were similar, with G2 even having one more member with good hygiene. These findings underscore the significance of the activities carried out on the first day, including maternal sensitivity, clinical examinations of the children, and the experimentation process in a simulated laboratory.

One of the limitations of the present study was the reduced number of participants in the group that underwent the face-to-face intervention due to lower attendance during the second meeting. Another limitation was related to the participants’ limited availability of time, which hindered the assessment of maternal oral health status and the collection of data in person. However, in order to mitigate potential biases during the phone calls, we established an information collection protocol and standardized the process. A limitation in terms of the qualitative analysis was that there was no external researcher involved in handling the information, which, if there had been, could have reduced confirmation bias.

In addition, this study did not include a long-term follow-up to assess the oral hygiene and caries status of the children due to the loss of the population, as many children changed educational institutions. Therefore, it is recommended that this educational strategy be implemented in a larger population that allows for long-term follow-up.

Finally, after 6 months, some participants exhibited a loss of knowledge regarding the early sign of caries and the microbiological link between sugar consumption and dental caries. This highlights the need to incorporate an experimental reinforce these critical concepts within the strategy.

## Conclusions

The implementation of a strategy rooted in neuroeducation elements, including emotions, experimentation, and play, yielded highly beneficial outcomes for the participants. These benefits were evident in terms of the enhanced knowledge and scores related to oral health, better comprehension of caries etiology, improved attitudes toward oral care and oral hygiene for children. Nevertheless, further efforts are required to reinforce the understanding of the connection between bacteria, sugar, and dental caries.

### Supplementary Information

**Additional file 1: Appendix 1.** Telephone interview guide for the study.

**Additional file 2: Appendix A2.** Dental history of the children of the participating mothers or caregivers.

**Additional file 3: Appendix A3.** Distribution of participants’ answers about oral health knowledge.

## Data Availability

All data generated or analysed during this study are included in this published article and its supplementary information files.
