# Supplementary material for: Enhancing knowledge, attitudes, and practices related to dental caries in mothers and caregivers of children through a neuroeducational strategy
Source: BMC Oral Health. 2024 Jan 9;24:60. doi: 10.1186/s12903-023-03734-0 (PMC10775469; doi:10.1186/s12903-023-03734-0)
Supplement: Supplementary file 1 — Additional file 1: Appendix 1. Telephone interview guide for the study. [file 12903_2023_3734_MOESM1_ESM.docx]

**Appendix 1. Telephone interview guide for the study**

1. **Introduction**

Hello _________________, I want to thank you for taking the time to meet with me today. My name is ____________________________ and I would like to talk to you about your knowledge, attitudes, and practices on dental caries. I am calling you since you are interesting in participating in the study “Effect of a neuroeducational strategy to improve knowledge, attitudes, and practices on dental caries among mothers and caregivers of children”, and also signed the informed consent form once we explained the study in the past parents' meeting. As explained at the parents' meeting in the kindergarten, this study aims to determine the effect of a neuroeducational strategy explaining the etiology of cavities in mothers of children aged 1 to 5 years.

The idea of this interview is to collect information that will allow us to measure the impact of the educational strategy after its implementation. For this reason, we will conduct this interview at three moments: before implementing the educative activities; immediately and 6 months after implementing them. During the first telephone call, and before the interview, we will ask questions about your sociodemographic information and, the clinical history of your child, and we will apply a questionnaire on knowledge, and we will finish with the interview. In the second and third interviews, we will only apply the knowledge questionnaire and end the interview.

Finally, it is necessary to tell you that the interviews should take approximately half an hour, and I will be done by telephone at a time that is convenient for you, just as we did this time. I will be recording the session, so please be sure to speak up so that we don’t miss your comments. Remember, all responses will be kept confidential, this means that your interview responses will only be shared with research team members and we will ensure that any information we include in our report does not identify you as the respondent. You don’t have to talk about anything you don’t want to, and you may end the interview at any time.

Are there any questions about what I have just explained? Are you willing to participate in this interview?

If you agree, we will start the interview, and for this, I will ask questions where you can answer what you know or do in your day to day.

1. **Open-ended questions concerning dental caries.**

1. ¿What do you know about dental cavities?

2. ¿Which elements or actions avoid dental cavities? ¿Which would be the most important?

3. ¿When do you know if your child/children has/have dental cavities?

4. ¿Do you know how sweet food and beverages act in the development of dental cavities?

5. ¿Who is responsible for the oral hygiene of your child/children?

6. ¿Is it important to take your child/children to the dental office? ¿When should you take your child/children to the dentist? ¿How often should you take your child/children to the dentist?

7. ¿When did you first take your child/children to the dentist?

8. ¿Does your child have dental anxiety?

9. ¿Who brushes your child's teeth? ¿How do you motivate your child/children to brush teeth? ¿Do you teach your child/children about dental care?

10. ¿Do you use toothpaste to brush your children's teeth? ¿How much toothpaste do you use?

**3. Closing**

Is there anything more you would like to add? We’ll be analyzing the information you and others gave me and submitting a report to the university, and we will do a scientific article in a few months.

Thank you for your time.
