# Supplementary material for: Enhancing knowledge, attitudes, and practices related to dental caries in mothers and caregivers of children through a neuroeducational strategy
Source: BMC Oral Health. 2024 Jan 9;24:60. doi: 10.1186/s12903-023-03734-0 (PMC10775469; doi:10.1186/s12903-023-03734-0)
Supplement: Supplementary file 2 — Additional file 2: Appendix A2. Dental history of the children of the participating mothers or caregivers. [file 12903_2023_3734_MOESM2_ESM.docx]

**Appendix A2.** Dental history of the children of the participating mothers or caregivers

| Dental history | GP1 | GP2 |
| --- | --- | --- |
| Visit to the dentist %(n) | | |
| Yes | 58.3 (7) | 95.5 (21) |
| No | 41.7 (5) | 4.5 (1) |
| Dental treatment %(n) | | |
| Yes | 41.7 (5) | 90.9 (20) |
| No | 16.6 (2) | 4.5 (1) |
| Not applicable | 41.7 (5) | 4.5 (1) |
| Type of treatment received %* (n) | | |
| Prophylaxis | 37.5 (6) | 39.2 (20) |
| Sealants | 6.25 (1) | 11.8 (6) |
| Fluorine | 37.5 (6) | 39.2 (20) |
| Operation | 12.5 (2) | 9.8 (5) |
| Exodontics | 0 | 0 |
| Maxillary orthopedics | 6.25 (1) | 0 |
| Previous experience with dentist %(n) | | |
| Good | 41.7 (5) | 72.7 (16) |
| Regular | 8.3 (1) | 18.2 (4) |
| Bad | 8.3 (1) | 4.5 (1) |
| No experience | 41.7 (5) | 4.5 (1) |
| Last visit to the dentist %(n) | | |
| 1 day-6 months | 16.7 (2) | 22.7 (5) |
| 7-12 months | 8.33 (1) | 50 (11) |
| 13-24 months | 25 (3) | 13.6 (3) |
| More than 24 months | 8.33 (1) | 4.5 (1) |
| Does not remember | 0 | 4.5 (1) |
| None | 41.7 (5) | 4.5 (1) |
| Frequency of tooth brushing %(n) | | |
| 1 time per day | 0 | 13.6 (3) |
| 2 times a day | 58.3 (7) | 50 (11) |
| 3 times a day | 41.7 (5) | 36.6 (8) |
| Never | 0 | 0 |
| Oral hygiene items %(n) | | |
| Brushing with toothpaste | 100 (12) | 72.7 (16) |
| Brushing, toothpaste, and dental floss | 0 | 13.6 (3) |
| Brushing, toothpaste, and dental rinse | 0 | 13.6 (3) |
| None | 0 | 0 |
| Frequency of consumption of sweets and flour %(n) | | |
| Never | 0 | 0 |
| 1-3 times a day | 75 (9) | 86.4 (19) |
| 4-6 times a day | 25 (3) | 13.6 (3) |
| >6 times a day | 0 | 0 |
| Hard tissues %(n) | | |
| Healthy | 16.7 (2) | 36.4 (8) |
| Dental caries | 50 (6) | 54.5 (12) |
| Other alteration | 33.3 (4) | 9.09 (2) |

%* Percentage calculated according to total treatments
