# Supplementary material for: Enhancing knowledge, attitudes, and practices related to dental caries in mothers and caregivers of children through a neuroeducational strategy
Source: BMC Oral Health. 2024 Jan 9;24:60. doi: 10.1186/s12903-023-03734-0 (PMC10775469; doi:10.1186/s12903-023-03734-0)
Supplement: Supplementary file 3 — Additional file 3: Appendix A3. Distribution of participants’ answers about oral health knowledge. [file 12903_2023_3734_MOESM3_ESM.docx]

Appendix A3. Distribution of participants’ answers about oral health knowledge.

| Questions | Answers | G1 (n=12) | | | G2 (n=22) | | |
| --- | --- | --- | --- | --- | --- | --- | --- |
|  |  | **Before**  **% (n)** | **Immediate impact**  **% (n)** | **Impact after six months**  **% (n)** | **Before**  **% (n)** | **Immediate impact**  **% (n)** | **Impact after six months**  **% (n)** |
| 1. Why do dental cavities occur? | a. Heritage | 8.3 (1) | 8.3 (1) | 0 | 9.1 (2) | 4.5 (1) | 0 |
|  | b. Eating sweets/poor diet* | 91.7 (11) | 83.3 (10) | 66.7 (8) | 77.3 (17) | 90.9 (20) | 72.7 (16) |
|  | Poor teeth cleaning/bacteria* | 100 (12) | 100 (12) | 100 (12) | 90.9 (20) | 100 (22) | 95.5 (21) |
| 2. In your opinion, what is the best way to prevent tooth decay? | Attending the dentist* | 75 (9) | 75 (9) | 75 (9) | 81.8 (18) | 90.9 (20) | 86.4 (19) |
|  | With proper teeth cleaning* | 100 (12) | 83.3 (10) | 100 (12) | 81.8 (18) | 90.9 (20) | 95.5 (21) |
|  | Avoiding sweets consumption* | 91.7 (11) | 91.7 (11) | 66.7 (8) | 68.2 (15) | 86.4 (19) | 81.8 (18) |
| 3. In your opinion, what is the main function of toothbrushing? | Removing bacteria from the teeth (bacterial plaque)* | 83.3 (10) | 91.7 (11) | 75 (9) | 81.8 (18) | 90.9 (20) | 90.9 (20) |
|  | Remove the food* | 75 (9) | 91.7 (11) | 83.3 (10) | 86.4 (19) | 77.3 (17) | 77.3 (17) |
|  | Feeling clean mouth | 75 (9) | 75 (9) | 42 (5) | 81.8 (18) | 59.1 (13) | 63.6 (14) |
| 4. Is it important to take care of baby (primary) teeth? (If yes, go to the next question, if no, go to question 6). | Yes* | 100 (12) | 100 (12) | 100 (12) | 95.5 (21) | 95.5 (21) | 95.5 (21) |
| 5. What is the most important reason to take care of the baby teeth? | Having a beautiful smile | 50 (6) | 50 (6) | 25.0 (3) | 59.1 (13) | 31.8 (7) | 45.5 (10) |
|  | Have good dental and general health* | 100 (12) | 100 (12) | 100 (12) | 95.5 (21) | 90.9 (20) | 95.5 (21) |
|  | To be able to eat and talk well | 58.3 (7) | 91.7 (11) | 25.0 (3) | 63.6 (14) | 63.6 (14) | 54.5 (12) |
| 6. Why not? | Because these teeth are replaced by permanent teeth. | 0 | 0 | 0 | 0 | 4.5 (1) | 0 |
|  | Because when the child is small, it is not necessary | 0 | 0 | 0 | 4.5 (1) | 0 | 0 |
|  | Because they are easily damaged | 0 | 0 | 0 | 0 | 0 | 0 |
| 7. Have you been told how to care for your child’s teeth? (If yes, go to the next question, if no, go to question 9) | Yes* | 58.3 (7) | 91.7 (11) | 100 (12) | 77.3 (17) | 100 (22) | 95.5 (21) |
| 8. Who has told you how to care for your child’s teeth? | Doctor/Nurse | 16.7 (2) | 25.0 (3) | 0 | 18.2 (4) | 22.7 (5) | 4.5 (1) |
|  | Dentist* | 41.7 (5) | 83.3 (10) | 100 (12) | 72.7 (16) | 86.4 (19) | 90.9 (20) |
|  | Community Mothers | 0 | 0 | 0 | 0 | 0 | 0 |
| 9. When does the child start teething? | From birth | 8.3 (1) | 16.7 (2) | 0 | 31.8 (7) | 18.2 (4) | 22.7 (5) |
|  | During the first year* | 91.7 (11) | 91.7 (11) | 100 (12) | 90.9 (20) | 95.5 (21) | 95.5 (21) |
|  | During the second year | 16.7 (2) | 8.3 (1) | 0 | 0 | 9.1 (2) | 13.6 (3) |
| 10. When to start cleaning the child's mouth? | From the moment the child is born* | 41.7 (5) | 66.7 (8) | 91.7 (11) | 72.7 (16) | 86.4 (19) | 77.3 (17) |
|  | From the appearance of milk teeth (temporary) | 66.7 (8) | 41.7 (5) | 33.3 (4) | 31.8 (7) | 22.7 (5) | 22.7 (5) |
|  | From the appearance of permanent teeth | 25.0 (3) | 16.7 (2) | 8.3 (1) | 9.1 (2) | - 1. (2) | 0 |
| 11. Why is it necessary to perform hygiene of the child's mouth/teeth? | To prevent cavities | 75.0 (9) | 66.7 (8) | 8.3 (1) | 63.6 (14) | 45.5 (10) | 9.1 (2) |
|  | To create habit | 58.3 (7) | 50 (6) | 0 | 50 (11) | 36.4 (8) | 0 |
|  | To create habit and to prevent cavities* | 83.3 (10) | 83.3 (10) | 91.7 (11) | 86.4 (19) | 95.5 (21) | - 1. (19) |
| 12. In your opinion, is toothpaste necessary for cleaning teeth? (If yes, go to the next question, if no, go to question 14) | Yes* | 91.7 (11) | 91.7 (11) | 100 (12) | 81.8 (18) | 86.4 (19) | 90.9 (20) |
| 13. What is the most important thing about toothpaste? | It leaves teeth clean and white | 83.3 (10) | 75 (9) | 33.3 (4) | 72.7 (16) | 45.5 (10) | 45.5 (10) |
|  | That gives a fresh breath | 66.7 (8) | 75 (9) | 33.3 (4) | 54.5 (12) | 59.1 (13) | 36.4 (8) |
|  | Allowing more fluoride in the mouth* | 50 (6) | 91.7 (11) | 75 (9) | 50 (11) | 77.3 (17) | 77.3 (17) |
| 14. When to start using toothpaste the children? | From the moment the child is born | 8.3 (1) | 0 | 0 | 4.5 (1) | 9.1 (2) | 0 |
|  | From the appearance of milk teeth (temporary)* | 100 (12) | 91.7 (11) | 100 (12) | 95.5 (21) | 95.5 (21) | 95.5 (21) |
|  | From the appearance of permanent teeth | 8.3 (1) | 16.7 (2) | 0 | 4.5 (1) | 9.1 (2) | 0 |
| 15. Is it necessary to control the amount of toothpaste used to clean the child's teeth? (If yes, go to the next question, if no, go to question 17) | Yes* | 100 (12) | 100 (12) | 100 (12) | 95.5 (21) | 95.5 (21) | - 1. (20) |
| 16. Why is it necessary to control the amount of toothpaste used to clean the child's teeth? | Because a lot of it makes the mouth fill with foam | 66.7 (8) | 33.3 (4) | 33.3 (4) | 50 (11) | 40.9 (9) | 45.5 (10) |
|  | Because many cause defects in permanent teeth* | 50 (6) | 83.3 (10) | 66.7 (8) | 36.4 (8) | 68.2 (15) | 59.1 (13) |
|  | Because a lot of it gives a spicy taste to the child. | 58.3 (7) | 33.3 (4) | 41.7 (5) | 63.6 (14) | 54.5 (12) | - 1. (10) |
| 17. Why not? | Because it does not cause any harm | 0 | 0 | 0 | 0 | 4.5 (1) | 0 |
|  | Because the greater the quantity, the cleaner the teeth are. | 0 | 0 | 0 | 0 | 0 | 0 |
|  | You have not been provided with information on the amount | 0 | 0 | 0 | 4.5 (1) | 0 | 4.5 (1) |
| 18. How much toothpaste should be use on the child's toothbrush? | Length of toothbrush | 25.0 (3) | 0 | 0 | 31.8 (7) | 22.7 (5) | 18.2 (4) |
|  | Half of the toothbrush length | 41.7 (5) | 8.3 (1) | 0 | 31.8 (7) | 22.7 (5) | 13.6 (3) |
|  | Length of the child's little fingernail* | 41.7 (5) | 100 (12) | 91.7 (11) | 45.5 (10) | 72.7 (16) | - 1. (18) |
| 19. In your opinion ¿What is fluoride used for on teeth? | To prevent and treat tooth decay* | 75.0 (9) | 83.3 (10) | 91.7 (11) | 86.4 (19) | 95.5 (21) | 90.9 (20) |
|  | To whiten the teeth | 50 (6) | 50 (6) | 16.7 (2) | 45.5 (10) | 40.9 (9) | 31.8 (7) |
|  | To leave fresh breath | 58.3 (7) | 58.3 (7) | 41.7 (5) | 40.9 (9) | 40.9 (9) | - 1. (9) |
| 20. By law, in Colombia ¿where is fluoride found? | In cooking salt and toothpaste | 25.0 (3) | 33.3 (4) | 8.3 (1) | 13.6 (3) | 31.8 (7) | 18.2 (4) |
|  | In drinking water, cooking salt and toothpastes* | 16.7 (2) | 66.7 (8) | 58.3 (7) | 22.7 (5) | 54.5 (12) | 40.9 (9) |
|  | In toothpastes only | 75.0 (9) | 16.7 (2) | 33.3 (4) | 68.2 (15) | 27.3 (6) | 36.4 (8) |
| 21. What does an initial caries lesion look like? | Like a gap in the tooth | 58.3 (7) | 16.7 (2) | 25.0 (3) | 36.4 (8) | 18.2 (4) | 36.4 (8) |
|  | Like a black spot on a tooth | 75.0 (9) | 25.0 (3) | 33.3 (4) | 90.9 (20) | 54.5 (12) | 50 (11) |
|  | Like a white spot on the tooth* | 25.0 (3) | 91.7 (11) | 75 (9) | 27.3 (6) | 63.6 (14) | 50 (11) |
| 22. In your opinion, ¿It is possible to prevent an initial dental caries lesion from turning into a cavity? (If yes, go to the next question, if no, go to question 24) | Yes* | 100 (12) | 100 (12) | 100 (12) | 95.5 (21) | 100 (22) | - 1. (21) |
| 23. What is the best way? | With good teeth cleaning and attendance to the dentist* | 100 (12) | 100 (12) | 100 (12) | 95.5 (21) | 100 (22) | 95.5 (21) |
|  | Stopping sweets and eating healthier | 75.0 (9) | 75 (9) | 58.3 (7) | 72.7 (16) | 72.7 (16) | 68.2 (15) |
|  | Using more fluoride toothpaste and rinses | 50 (6) | 83.3 (10) | 41.7 (5) | 54.5 (12) | 40.9 (9) | 54.5 (12) |
| 24. In your opinion, ¿Is it possible to prevent a cavity where a gap is visible from continuing to damage the tooth? (If yes, go to the next question). | Yes* | 75.0 (9) | 100 (12) | 91.7 (11) | 100 (22) | 100 (22) | 95.5 (21) |
| 25. What is the best way? | With good teeth cleaning and attendance to the dentist* | 75.0 (9) | 100 (12) | 91.7 (11) | 95.5 (21) | 95.5 (21) | 95.5 (21) |
|  | Covering the gap with nail polish or aspirin | 16.7 (2) | 25.0 (3) | 0 | 22.7 (5) | 13.6 (3) | 4.5 (1) |
|  | Having the tooth pulled out | 33.3 (4) | 33.3 (4) | 0 | 18.2 (4) | 4.5 (1) | 4.5 (1) |

*Correct answer
